# Adipokines and risk of rheumatoid arthritis: A two-sample multivariable Mendelian randomisation study

**DOI:** 10.1371/journal.pone.0286981

**Published:** 2023-06-09

**Authors:** Georgios K. Vasileiadis, Sergi Sayols, Sizheng Steven Zhao, Tahzeeb Fatima, Cristina Maglio

**Affiliations:** 1 Department of Rheumatology and Inflammation Research, Institute of Medicine, The Sahlgrenska Academy at University of Gothenburg, Gothenburg, Sweden; 2 Wallenberg Centre for Molecular and Translational Medicine at the University of Gothenburg, Gothenburg, Sweden; 3 Department of Medical Sciences, Molecular Epidemiology and Science for Life Laboratory, Uppsala University, Uppsala, Sweden; 4 CIBER Cardiovascular Diseases (CIBERCV), Instituto de Salud Carlos III, Madrid, Spain; 5 Division of Musculoskeletal and Dermatological Sciences, Centre for Genetics and Genomics Versus Arthritis, School of Biological Sciences, Faculty of Biology Medicine and Health, Manchester Academic Health Science Centre, The University of Manchester, Manchester, United Kingdom; 6 Department of Rheumatology, Sahlgrenska University Hospital, Gothenburg, Sweden; University of Bern: Universitat Bern, SWITZERLAND

## Abstract

Adiponectin, leptin, and resistin are thought to be involved in the pathogenesis of rheumatoid arthritis (RA). However, the causal relationship between these adipokines and the risk for RA is unclear. We performed a range of two-sample Mendelian randomisation (MR) analyses to assess the causal effect of circulating adiponectin, leptin, and resistin on RA risk in European and East Asian individuals. Different sets of adiponectin-, leptin-, and resistin-related genetic variants were used as instruments for genetically determined adipokine levels. As body mass index (BMI) is a risk factor for RA and affects adipokine levels, multivariable MR was used to calculate the causal effect of each adipokine on RA risk taking BMI into account. Several MR analyses revealed no evidence of a causal relationship between circulating adiponectin, leptin, or resistin levels and RA risk in either Europeans or East Asians. Similarly, multivariable MR did not provide evidence of any causal effect of adiponectin, leptin, or resistin on RA risk when taking BMI into account. This MR study shows for the first time that genetically determined levels of adiponectin, leptin, or resistin do not have a direct causal effect on the risk of developing RA after adjustment for BMI.

## Introduction

Adiponectin, leptin, and resistin are cytokines produced by the adipose tissue, so called adipokines, and they are thought to be involved in the pathogenesis of rheumatoid arthritis (RA), a chronic inflammatory disease [1, 2]. In fact, adiponectin, leptin, and resistin levels are increased in both blood and synovial fluid of patients with RA [3–7]. All three adipokines have been associated with markers of inflammation and disease activity in patients with RA [4, 8–11]. In addition, we previously showed that circulating adiponectin levels rise years before RA onset [12, 13]. All these epidemiological findings suggest that adiponectin, leptin and resistin could be involved in the initiation and progression of RA. However, a causal association between these adipokines and RA risk cannot be postulated from such epidemiological studies alone, as they might be biased by confounders and reverse causality.

Mendelian randomisation (MR) is an analytical method that aims to estimate if a certain exposure (in our case, adipokines) has a causal relationship with a certain outcome (in our case, RA) by using genetic variants for a given exposure as instrumental variables (IVs) [14]. A recent MR study conducted in Europeans demonstrated that circulating adiponectin levels are not causally associated with the risk of developing RA [15]. However, that study used univariate MR and did not consider body-mass index (BMI) as a confounder. This is a limitation because high BMI is a risk factor for RA and is also associated with lower circulating levels of adiponectin [16, 17]. Therefore, any analysis of the causal relationship between adiponectin and RA needs to account for the potential confounding role of BMI.

The purpose of this study was to determine whether circulating adiponectin, leptin, or resistin levels are causally associated with the risk of developing RA by using a two-sample MR approach. We also performed a multivariable MR to determine if adiponectin, leptin, or resistin have a causal effect on RA development independently of BMI.

## Materials and methods

### Data sources

IVs for adiponectin, leptin, resistin, and RA were derived from large Genome-wide association studies (GWASs). Specifically, we used data from the largest meta-analysis of GWASs in Europeans, involving 39,883 individuals [18], and from a meta-analysis of GWASs involving 18,079 individuals of East Asian ancestry [19] to construct genetic IVs for circulating adiponectin levels. Genetic variants related to circulating leptin levels were identified from a GWAS including a total of 52,140 individuals of European ancestry [20]. Furthermore, a genetic dataset for circulating resistin levels was built from data from a genome-wide meta-analysis involving 30,931 Europeans from the Systematic and Combined AnaLysis of Olink Proteins (SCALOP) consortium [21]. We obtained regression coefficients and corresponding standard errors for the association between adipokine-related single-nucleotide polymorphisms (SNPs) and the risk to develop RA for both European and East Asian populations from a three-stage trans-ethnic GWAS meta-analysis in a total of 80,799 subjects [22]. The meta-analysis evaluated ~10 million SNPs. All 29,880 RA cases (88.1% seropositive and 9.3% seronegative for anti-citrullinated peptide antibody [ACPA] or rheumatoid factor [RF], and 2.6% of unknown autoantibody status) fulfilled the 1987 criteria of the American College of Rheumatology for RA diagnosis or were diagnosed with RA by a rheumatologist [23]. All GWAS data were publicly available.

To run the multivariable MR analyses, publicly available summary statistics data of two separate BMI GWASs, in European and East Asian individuals, were used to obtain the information for IV-BMI associations. For Europeans, the information was extracted using the results from a meta-analysis of GWASs for BMI. The study combined the summary statistics of the Genetic Investigation of ANthropometric Traits (GIANT) consortium [24] with a BMI GWAS in the UK Biobank cohort and included around 700,000 individuals of European ancestry [25]. To obtain the IV-BMI association data in East Asians, the summary statistics data from a GWAS in Biobank Japan project including 173,430 individuals was used [26].

### Ethics statement

This study only used published or publicly available data and no original data were collected. Ethical approval by the corresponding institutional review board or ethics committee including information on participants’ written informed consent for each of the studies included here can be found in the original publications.

### Instrumental variables

Summary statistics data of ten and four adiponectin-related SNPs were derived, respectively, for European and East Asian populations from their respective studies (S1 Table) [18, 19]. All SNPs were associated with circulating adiponectin levels at genome-wide significance (p-value < 5×10^−8^) and were not in linkage disequilibrium (LD) with each other (r^2^ < 0.1). Variance explained (R^2^) was calculated using 2*EAF(1-EAF)β^2^, where EAF is the effect allele frequency and β the association estimate. F-statistic was derived using (R^2^/K)/[(1-R^2^)(N-K-1)], where K is the number of SNPs and N is the sample size. F-statistic > 10 is considered suggestive of adequate instrument strength [27]; all SNPs with F-statistics at or below 10 were excluded from the analysis. While the SNP rs2980879 fulfilled the selection criteria, it was excluded from the analyses for being palindromic. We built two sets of genetic instruments to perform two MR analyses for circulating adiponectin in the European population:

1. A liberal analysis, which consisted of all ten SNPs regardless of locus (S1 Table)
2. A conservative analysis, which only consisted of four SNPs within the *ADIPOQ* locus (±50 kb) (S1 Table). As *ADIPOQ* encodes adiponectin, we regarded this approach as unlikely to be biased by horizontal pleiotropy considering the functional relationship between *ADIPOQ* and circulating adiponectin levels.

For East Asians, a single set of instruments was used as IVs to perform liberal MR analysis, as there was only one SNP within the ADIPOQ locus, precluding a conservative MR analysis.

Four SNPs (S1 Table) were selected as IVs for circulating leptin levels, after exclusion of two palindromic SNPs, rs8043757 and rs10487505 [20]. All SNPs were associated with circulating leptin levels at p-value < 5×10^−5^, were not in LD with each other (r^2^ < 0.1), and had F-statistics >10.

Eleven SNPs derived from the European population were selected as IVs for circulating resistin levels (S1 Table) [21]. All SNPs were associated with circulating resistin levels at genome-wide significance (p-value < 5×10^−8^), were not in LD with each other (r^2^ < 0.1), and had F-statistics > 10. For Europeans, rs77691416 was replaced with a proxy SNP (rs17616841) in the outcome data at an r^2^ = 1 (S2 Table).

### Main MR analysis

We performed single SNP analysis for each of the selected SNPs, where MR estimates were calculated using the Wald ratio method [28]. The effect of each adipokine’s levels on RA risk was calculated using the inverse-variance weighted (IVW) method, with a random-effects model. The IVW method is considered the most efficient as it carries the greatest statistical power [29]. However, IVW is subject to bias, since it assumes the absence of any horizontal pleiotropy i.e., the effects of the genetic instruments on the considered outcome through pathways independent of the exposure [29]. To address this, we performed several sensitivity analyses with more flexible assumptions on horizontal pleiotropy. To account for multiple testing in three adipokines to RA causality measurements, we used Bonferroni correction (0.05/3). A p-corrected of 0.016 was considered statistically significant to indicate a causal effect.

### Sensitivity analysis

We estimated the intercept and slope of MR-Egger regression, where intercept represents the average horizontal pleiotropy while slope indicates a pleiotropy-adjusted MR estimate. A statistically significant intercept value (p < 0.05) indicates the presence of horizontal pleiotropy [30]. In addition, the weighted median-based method was utilized, which measures the weighted median effect of all MR estimates produced by individual instruments, with weights equal to the inverse of the standard error. Finally, to identify whether a single genetic variant is driving the association between exposure and outcome, leave-one-out analysis was executed. In a leave-one-out analysis, MR is performed by leaving each SNP out of the analysis in turn. All analyses were conducted using the ‘MendelianRandomization’ package (v 0.5.0) in R (v 4.0.2) [31, 32].

### Multivariable MR analysis

We applied multivariable MR to assess if BMI modulates the causal effect of adiponectin, leptin or resistin on RA risk by adding BMI as an adjusting factor in the model. Multivariable MR is an extension of MR that deals with genetic variants that are associated with multiple risk factors [33]. It accounts for the potential pleiotropic effect of all exposures included in the analysis. To run this analysis, summary statistics data for all the IVs for adiponectin, leptin, and resistin were obtained from two separate GWASs for BMI [24, 25]. As multivariable MR requires all instruments to be associated with at least one exposure in the regression model, initially all variants were chosen to be included in the analysis. However, as the variant-BMI association data were missing for certain SNPs, these were either replaced by proxies at high LD (r^2^ ≥ 0.99), where available (S2 Table), or excluded from the final analyses. Regarding adiponectin in Europeans, rs1108842 was excluded from the final analysis, as variant-BMI association data were missing for this SNP and no proxies could be identified, leaving the instrument list to a count of 9. Concerning resistin in Europeans, four SNPs were excluded (rs10103048, rs3745367, rs73008259 and rs7589428) while three SNPs were replaced with proxies: rs3087852 replaced with rs8075668, at r^2^ = 0.99; rs4134826 replaced with rs4134831, at r^2^ = 1; rs6775731 replaced with rs2010527, at r^2^ = 1. In all populations, separate multivariable MR analyses were done using a ‘weighted regression‐based method’ approach that applies the IVW to a multivariable regression model [33]. Considering the selected set of instrument variants were uncorrelated, a random effect model was implied. Similar to the univariate MR, we also performed MR-Egger analysis for multivariable MR as a sensitivity analysis using multivariable weighted linear regression method [34]. All analyses were conducted using the ‘MendelianRandomization’ package (v 0.5.0) in R (v 4.0.2) [31, 32].

## Results

### No causal effect of adiponectin on the risk for RA

**Fig 1:**
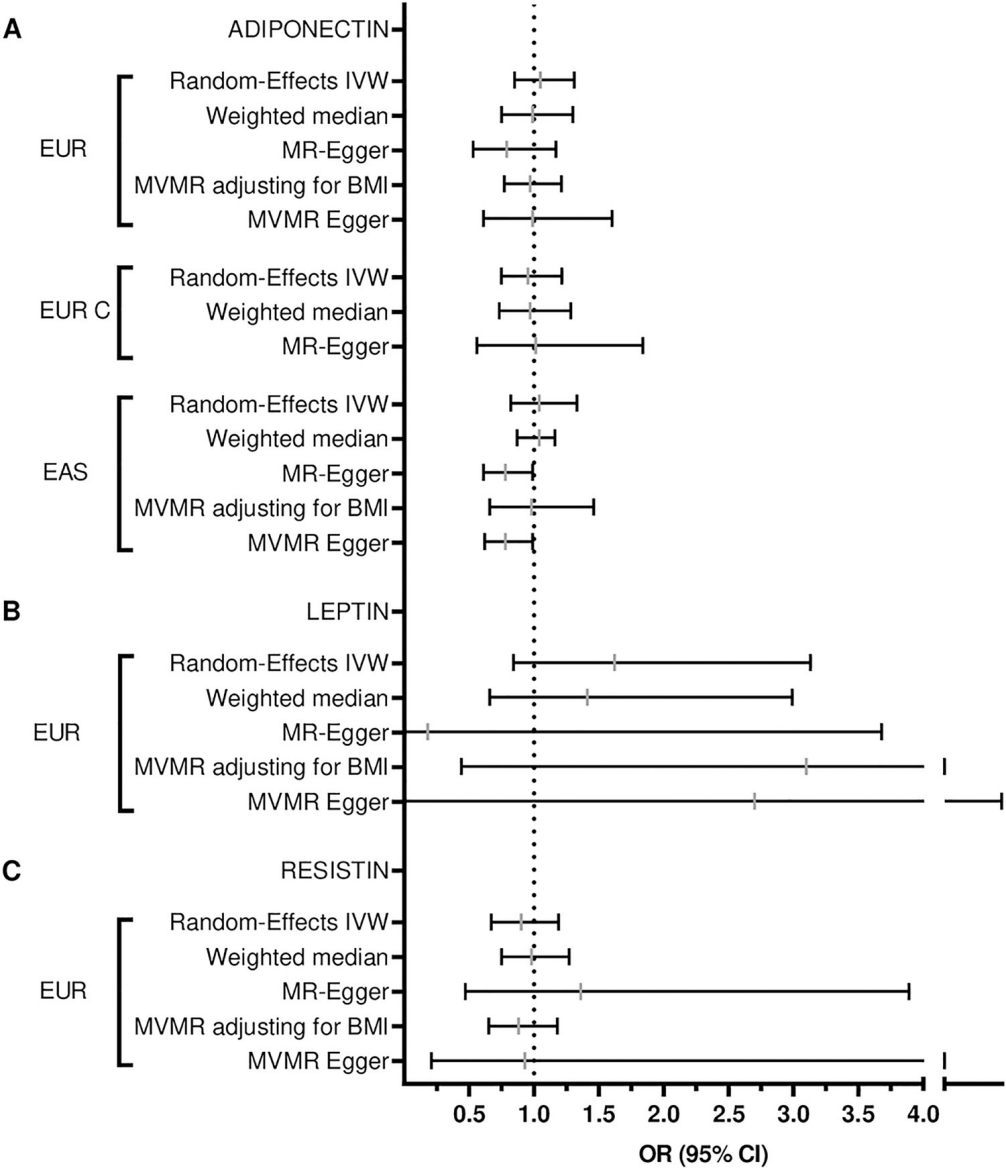
Forest plot of the causal effects of (A) adiponectin-, (B) leptin-, or (C) resistin-associated single nucleotide polymorphisms (SNPs) on rheumatoid arthritis (RA) risk. Shown are Mendelian randomisation (MR) results for Europeans (EUR) and East Asians (EAS). EUR C signifies a conservative approach for adiponectin to RA risk MR. An odds ratio (OR) is a measure of association between an exposure and an outcome, in which the OR represents the likelihood that an outcome will occur given a particular exposure, compared to the likelihood of the outcome occurring in the absence of that exposure. Statistical analyses were performed with the use of inverse-variance weighted (IVW) estimate, MR-Egger regression analysis and weighted median estimator. Multivariable MR (MVMR) was used to calculate the effect of adiponectin, leptin, and resistin to RA risk after adjustment for body mass index (BMI).

To determine if circulating levels of adiponectin were causally associated with the risk for RA, we first performed a univariate MR analysis in both Europeans and East-Asians. In Europeans, neither a liberal (odds ratio, OR = 1.05; 95% confidence interval, CI = 0.85–1.31; p = 0.65) nor a conservative approach (OR = 0.95; 95% CI = 0.75–1.22; p = 0.70, Table 1 and Fig 1A) revealed evidence of a causal effect of circulating adiponectin levels on RA risk. Similarly, adiponectin did not affect the risk of developing RA in East Asians (OR = 1.04; 95% CI = 0.82–1.33; p = 0.74; Table 1 and Fig 1A). Sensitivity analyses confirmed this lack of effect (Table 1, Fig 1A, S1 Fig).

**Table 1:** MR estimates from each method for assessing the causal effect of adiponectin, leptin, and resistin on the risk of RA in Europeans and East Asians.

**Adiponectin**					
Liberal analysis in Europeans					
*MR method*	*SNPs*	*β*	*SE*	*OR (95% CI)*	*p*
Random-effects IVW	10	0.050	0.11	1.05 (0.85–1.31)	0.65
Weighted median	10	-0.011	0.14	0.99 (0.75–1.30)	0.94
Egger intercept	10	0.021	0.01	1.02 (1.00–1.04)	0.09
MR-Egger	10	-0.235	0.20	0.79 (0.53–1.17)	0.24
MVMR adjusting for BMI	9	-0.032	0.12	0.97 (0.77–1.21)	0.78
MVMR Egger	9	-0.008	0.24	0.99 (0.61–1.60)	0.97
Conservative analysis in Europeans					
*MR method*	*SNPs*	*β*	*SE*	*OR (95% CI)*	*p*
Random-effects IVW	4	-0.048	0.12	0.95 (0.75–1.22)	0.70
Weighted median	4	-0.031	0.14	0.97 (0.73–1.28)	0.83
Egger intercept	4	-0.006	0.03	0.99 (0.94–1.05)	0.83
MR-Egger	4	0.014	0.30	1.01 (0.56–1.84)	0.96
East Asians					
*MR method*	*SNPs*	*β*	*SE*	*OR (95% CI)*	*p*
Random-effects IVW	4	0.041	0.12	1.04 (0.82–1.33)	0.74
Weighted median	4	0.006	0.08	1.01 (0.87–1.16)	0.94
Egger intercept	4	0.065	0.02	1.07 (1.02–1.12)	0.004
MR-Egger	4	-0.249	0.12	0.78 (0.61–0.99)	0.042
MVMR adjusting for BMI	4	-0.017	0.20	0.98 (0.66–1.46)	0.93
MVMR Egger	4	-0.250	0.12	0.78 (0.62–0.99)	0.044
**Leptin**					
*MR method*	*SNPs*	*β*	*SE*	*OR (95% CI)*	*p*
Random-effects IVW	4	0.481	0.34	1.62 (0.84–3.13)	0.15
Weighted median	4	0.340	0.39	1.41 (0.66–2.99)	0.38
Egger intercept	4	0.067	0.16	1.07 (0.79–1.45)	0.66
MR-Egger	4	-1.710	5.06	0.18 (0.00–3.68)	0.74
MVMR adjusting for BMI	4	1.132	1.00	3.10 (0.44–21.93)	0.26
MVMR Egger	4	0.993	7.12	2.70 (0.00–3,078,644)	0.89
**Resistin**					
*MR method*	*SNPs*	*β*	*SE*	*OR (95% CI)*	*p*
Random-effects IVW	11	-0.109	0.15	0.90 (0.67–1.19)	0.46
Weighted median	11	-0.025	0.13	0.98 (0.75–1.27)	0.85
Egger intercept	11	-0.033	0.04	0.97 (0.89–1.05)	0.42
MR-Egger	11	0.304	0.54	1.36 (0.47–3.89)	0.57
MVMR adjusting for BMI	7	-0.134	0.15	0.88 (0.65–1.18)	0.38
MVMR Egger	7	-0.070	0.75	0.93 (0.21–4.06)	0.93

Abbreviations: MR, Mendelian randomisation; RA, rheumatoid arthritis; SNP, single nucleotide polymorphism; SE, standard error; OR, odds ratio; 95% CI, 95% confidence interval; IVW, inverse-variance weighted; MVMR, multivariable MR; BMI, body mass index.

In Europeans, directional pleiotropy was found to be unlikely to bias the results, as shown by the Egger intercept in both liberal (p = 0.09) and conservative analyses (p = 0.83), suggesting reliability of the MR estimates. Conversely, directional pleiotropy was detected in East Asians, as shown by the Egger intercept (p = 0.004, Table 1). In addition, the leave-one-out analysis suggested that the main MR outcome in East Asians was driven by a single SNP, rs4783244 (S1 Fig).

We then performed a multivariable analysis by taking BMI into account in the model. The results of the univariate analysis were confirmed by multivariable MR, which showed no causal effect of adiponectin on RA in both Europeans (OR = 0.97; 95% CI = 0.77–1.21; p = 0.78) and East Asians (OR = 0.98; 95% CI = 0.66–1.46; p = 0.93. Table 1 and Fig 1A). The estimates provided by multivariable MR were comparable with the previous univariate MR analyses.

### No causal effect of leptin on the risk for RA

Next, we tested if leptin was causally linked to RA. The analysis was limited to individuals of European ancestry as no sufficiently large genetic studies on leptin levels in East Asians were found. No causal effect of circulating leptin on RA risk in Europeans was found by the univariate MR analysis (OR = 1.62; 95% CI = 0.84–3.13; p = 0.15) nor by subsequent sensitivity analyses (Table 1, Fig 1B and S1 Fig). Directional pleiotropy was unlikely to bias the results, as Egger intercept was not significant (p = 0.66), adding reliability to the MR estimates. Additionally, the multivariable MR analysis accounting for BMI did not provide evidence of a causal effect of leptin on the risk for RA in Europeans (OR = 3.10; 95% CI = 0.44–21.93; p = 0.26; Table 1 and Fig 1B).

### No causal effect of resistin on the risk for RA

Finally, we determined if resistin was causally linked to the risk for RA. This analysis was also limited to Europeans due to lack of sufficiently large genetic studies on resistin levels in East Asian individuals. Univariate MR analysis did not indicate a causal effect of circulating resistin on RA risk (OR = 0.90; 95% CI = 0.67–1.19; p = 0.46; Table 1 and Fig 1C). Sensitivity analysis confirmed no causality between resistin levels and RA risk (Table 1, Fig 1C and S1 Fig). In addition, Egger intercept (p = 0.42) suggested the absence of directional pleiotropy in our datasets. Furthermore, multivariable MR estimates did not support a causal effect of circulating resistin levels on RA risk when taking BMI into account (OR = 0.88; 95% CI = 0.65–1.18; p = 0.38).

## Discussion

This two-sample MR study does not support a direct causal effect of circulating adiponectin, leptin, or resistin levels on the development of RA. The lack of effect was further confirmed by multivariable MR analyses, which took BMI into account.

Although both epidemiological and *in vitro* studies have linked adiponectin to the pathogenesis of RA, it was unknown if adiponectin has a direct causal effect on RA development [8, 9, 12, 35]. Recently, a univariate MR study in Europeans found that circulating levels of adiponectin do not have a causal effect on RA risk [15]. However, this study did not take BMI into account. High BMI is a risk factor for RA and is associated with lower circulating adiponectin levels. Thus, BMI is a confounder when studying the association between adiponectin and RA risk. Therefore, after the univariate MR analysis showed no causal link between adiponectin and RA risk, we decided to perform a multivariable MR to account for possible shared genetic background between circulating adiponectin and BMI. Multivariable MR is a method that allows estimation of the direct causal effects of each risk factor independently in a single analysis model [29, 33]. In our study, the multivariable MR analysis showed that circulating adiponectin is not causally linked to RA risk regardless of BMI.

Both leptin and resistin are increased in blood and synovial fluid of patients with RA compared to controls and they can induce the production of pro-inflammatory cytokines in fibroblast-like synoviocytes collected from patients with RA [3–6, 36–38]. However, our univariate MR analysis found that circulating leptin and resistin do not have a causal effect on the risk to develop RA. Since both circulating leptin and resistin levels are affected by BMI, we also performed a multivariable MR accounting for BMI. Multivariable MR confirmed the lack of effect of those adipokines on RA risk independently of BMI.

Our study focused on the effect of adipokines on the risk to develop RA, without any examination of disease progression, response to treatment, or comorbidities. Even if this MR study did not identify a causal link between adiponectin, leptin, or resistin and the risk of RA, it does not rule out that these adipokines might be involved in the pathogenesis of the disease, potentially by contributing to inflammation [4, 8–10]. Furthermore, they could also have a role as markers of RA activity or response to anti-rheumatic treatment, as suggested by previous results [39–41]. Consequently, despite our negative result on the causal effect of adiponectin, leptin, and resistin on RA risk, the role of these adipokines in inflammation and immunomodulation is well documented and, thereby, not challenged by this study.

MR studies are powerful tools to assess causality between a certain exposure and a defined outcome. While observational studies are inherently likely to suffer from bias due to residual confounding or reverse causality, MR minimizes these possibilities. Nevertheless, MR is still susceptible to bias from the association of genetic variants with more than one outcome, an occurrence called pleiotropy. To avoid pleiotropy, we have not only performed a multivariable MR accounting for the confounding effect of BMI, but also a variety of sensitivity analyses. Another strength of this study is that we have included large cohorts of subjects with different genetic backgrounds, giving our study significant power to detect any causality.

This study is not without limitations. Only a small number of SNPs were selected as IVs in our analyses. Moreover, most genetic variants have a relatively limited effect on a particular exposure, e.g., circulating adipokine levels, as they might only explain a small proportion of variance in that exposure (R^2^ (%) range: 0.03–5.02). It is important to note that there are other factors besides BMI that can affect adipokine levels, such as diet, exercise, and lipid profile [42–45]. However, BMI is the main determinant as it is linked to the aforementioned factors. Moreover, BMI is easy to measure in large cohorts, and there is abundant availability of GWAS data on it. The selection of BMI was also based on its primary relevance as a known risk factor for RA [16]. In addition, adiponectin circulates in three main forms, high, middle, and low molecular weight adiponectin. The GWAS studies on adiponectin only measured total adiponectin levels in blood, without any information given on the levels of particular forms. It is unknown from these studies if any particular form of adiponectin is associated with specific SNPs. Of note, Egger intercept for circulating adiponectin in the East Asian population was significantly larger than zero, indicating the presence of pleiotropic genetic variants in this dataset. As evident from the leave-one-out analysis, a single SNP, rs4783244, was driving the main MR outcome in East Asians. However, rs4783244 is a valid instrument and its nearest gene is *CDH13* that encodes T-cadherin, a receptor for adiponectin, strengthening the biological link between the SNP and the exposure [46]. Ultimately, the MR results are unlikely to be caused by invalid instruments as IVW, the method carrying the greatest statistical power, as well as weighted median and MR-Egger did not support a causal association between adiponectin and RA in East Asians.

In conclusion, the results of the MR analysis show for the first time, to the best of our knowledge, that circulating adiponectin, leptin, or resistin levels do not have a direct causal effect on the risk of developing RA after adjustment for BMI. Therefore, the association of these adipokines’ levels with RA, as suggested by epidemiological studies, does not appear to be causal.

## Supporting information

S1 Fig Leave-one-out sensitivity analysis. Each point on the y axis represents the inverse variance weighted (IVW) method applied to estimate the causal effect of **(A)** adiponectin in Europeans, **(B)** adiponectin in East Asians, **(C)** leptin in Europeans, or **(D)** resistin in Europeans on rheumatoid arthritis risk, excluding that particular variant from the analysis. The final point of each plot depicts the IVW estimate of the main analysis using all genetic variants.
(PDF)

S1 Table Main SNPs. (XLSX)

S2 Table Proxies. (XLSX)
