# Supplementary material for: Adipokines and risk of rheumatoid arthritis: A two-sample multivariable Mendelian randomisation study
Source: PLoS One. 2023 Jun 9;18(6):e0286981. doi: 10.1371/journal.pone.0286981 (PMC10256188; doi:10.1371/journal.pone.0286981)
Supplement: S1 Fig — Each point on the y axis represents the inverse variance weighted (IVW) method applied to estimate the causal effect of (A) adiponectin in Europeans, (B) adiponectin in East Asians, (C) leptin in Europeans, or (D) resistin in Europeans on rheumatoid arthritis risk, excluding that particular variant from the analysis. The final point of each plot depicts the IVW estimate of the main analysis using all genetic variants. (PDF) [file pone.0286981.s001.pdf]

Supplementary Figure 1

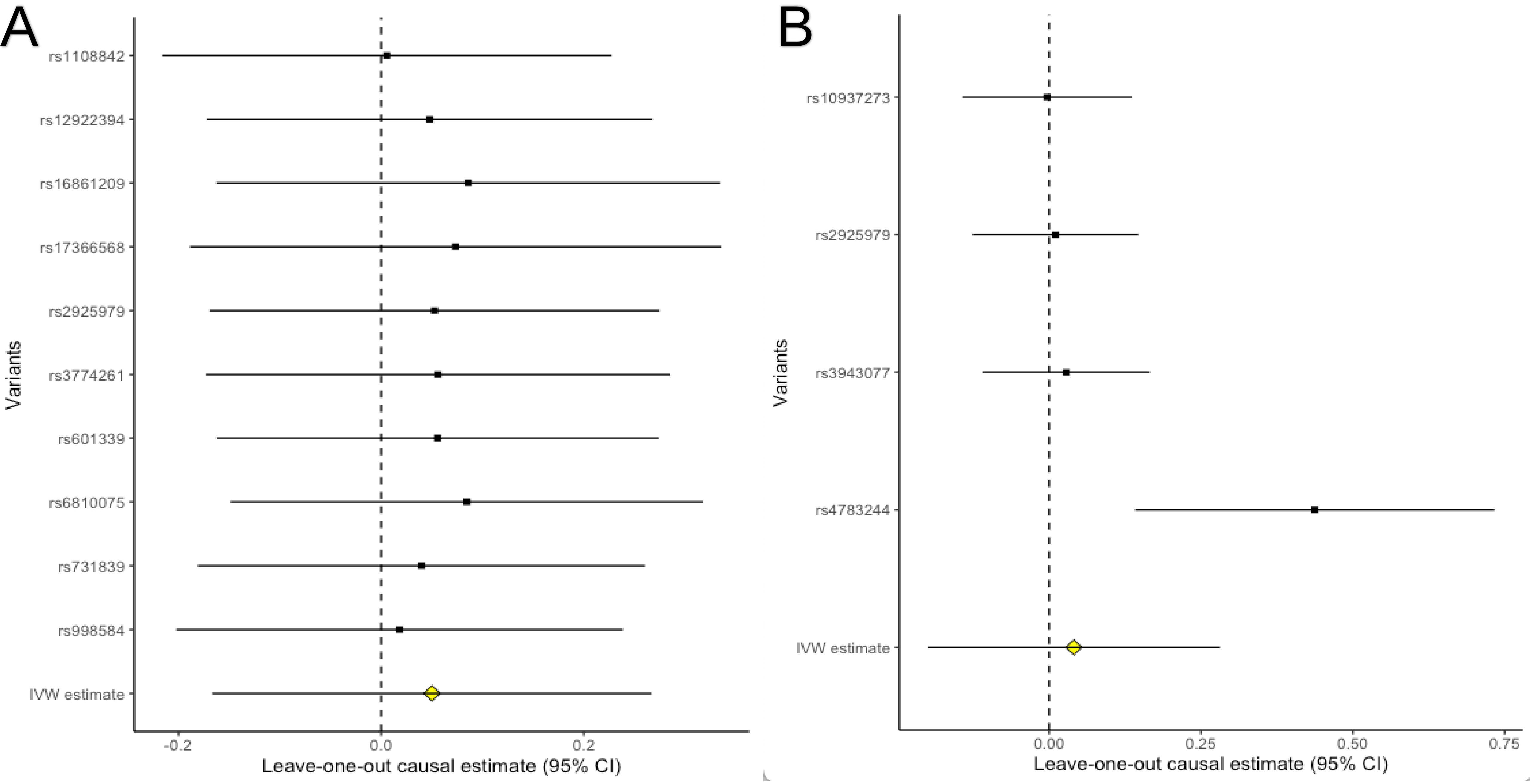

Supplementary Figure 1

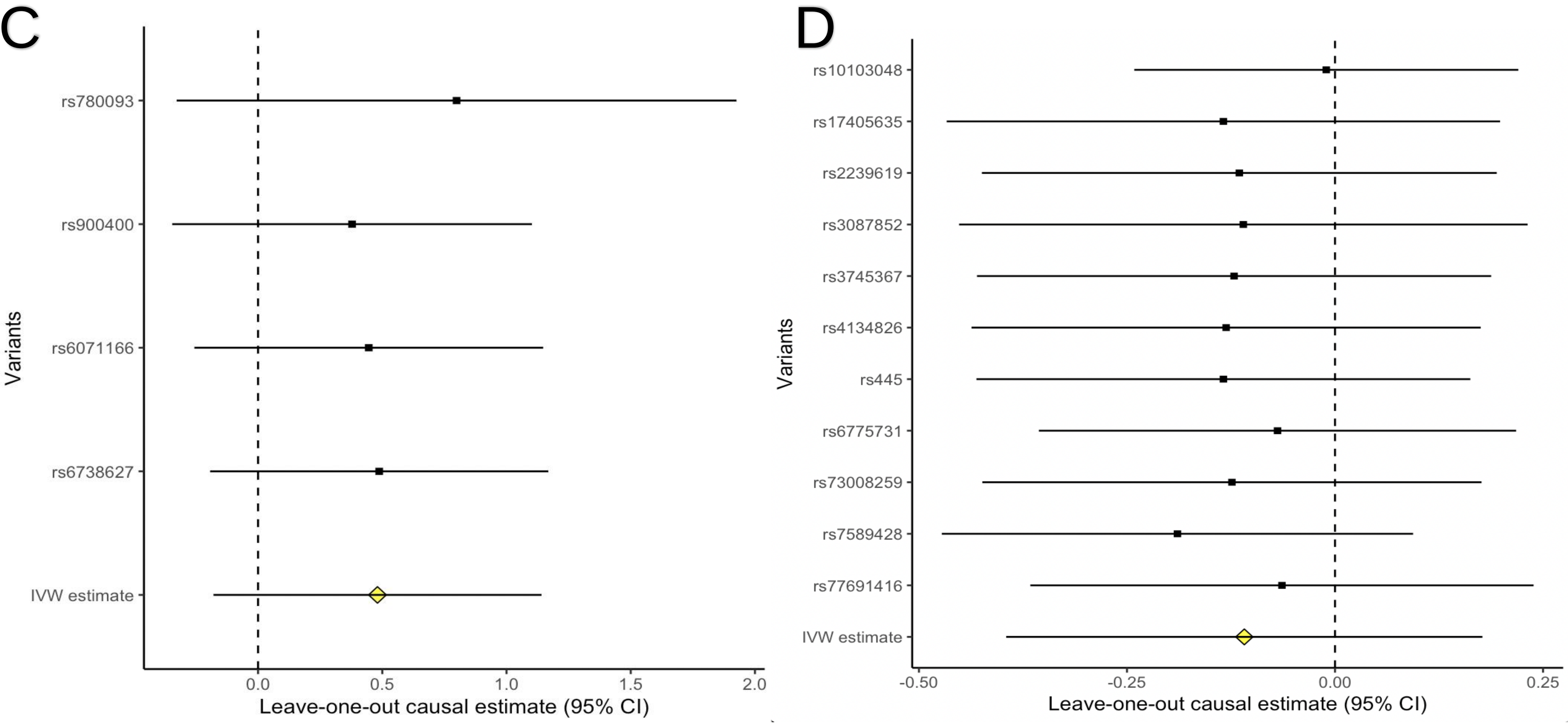

S1 Fig: Leave-one-out sensitivity analysis. Each point on the y axis represents the inverse variance weighted (IVW) method applied to estimate the causal effect of **(A)** adiponectin in Europeans, **(B)** adiponectin in East Asians, **(C)** leptin in Europeans, or **(D)** resistin in Europeans on rheumatoid arthritis risk, excluding that particular variant from the analysis. The final point of each plot depicts the IVW estimate of the main analysis using all genetic variants.
